# Opening Pandora’s Box: Person-Sensitive Conceptualization and Measurement in Psychological Science

**DOI:** 10.17505/jpor.2024.26271

**Published:** 2024-05-23

**Authors:** Artur Nilsson

**Affiliations:** 1 Department of Psychosocial Science, University of Bergen, Bergen, Norway; 2 Department of Behavioural Sciences and Learning, Linköping University, Linköping, Sweden

**Keywords:** persons, populations, psychological science, integrative framework

## Abstract

Although distinctions between the study of persons, the study of populations, and the study of mechanisms are helpful for illuminating mismatches between research assumptions, problems, and methods, it may be difficult to construe these as entirely discrete branches of psychological science. I suggest that it is more appropriate to view person-levelness (or person-sensitivity) as an ideal we should actively aspire toward, within the constraints placed by other goals such as generalizability and feasibility, when pursuing knowledge about individuals. It is an ideal that we can never hope to perfectly realize—the degree to which it is realized will always be a matter of degree, and there is therefore no clear line of demarcation between the person level and other branches of psychology. This ideal can nonetheless stimulate more person-sensitive conceptualizations, measurements, and analyses.

## Introduction

Psychology is an enormously complex science, encompassing a plethora of sub-disciplines and research traditions that intersect in complicated ways. Conceptualizing it in its entirety might seem like an impossible task, at least insofar as we are trying to provide a *descriptive* account of the kinds of research those of us who call ourselves psychological scientists are engaged in. Another approach is to provide a philosophically grounded *normative* account that focuses on the types of psychological knowledge that can (and should) be pursued and the proper methods for doing so. Such an approach has multiple advantages. It not only helps us to make sense of a vast and seemingly chaotic field but, perhaps more importantly, it stimulates critical thinking with respect to existing methodological practices and creativity in the development of novel theories and methods. In other words, it provides an impetus for improving psychological science and counteracts reification of existing structures and practices.

In the target article, Lundh (2023) makes a valuable contribution in this spirit, by setting forth a normative framework that divides psychology into three branches based on the types of research questions they address: person psychology, population psychology, and mechanism psychology. Synthesizing a burgeoning literature on person-oriented research, he provides ample illustration of mismatches between research questions and the methods that are commonly employed to address these questions—mismatches that frequently go either unnoticed or ignored, even when they are blatant (for a particularly striking example from political psychology, see Nilsson, 2024)—while also touching on the development of novel methods that are better tailored to the person level (e.g., Molenaar & Campbell, 2009). A particularly intriguing suggestion is that we need new kinds of psychotherapy research that dig into what is going on at the level of the individual to understand when and why psychotherapy works. For example, instead of assuming that the real-life implementations of complex treatment packages correspond in a simple and uniform way to their abstract labels (e.g., “cognitive behavior therapy”), we might need to study therapeutic skills “in action” at the person level, Lundh points out.

Although I am sympathetic to the distinctions between person, population, and mechanism research, it is not clear to me how we should think about the relations between these levels. While research questions are easy enough to fit into this classificatory scheme, it is a lot more difficult to unequivocally place all legitimate varieties of psychological studies into one of the three branches. In particular, the seemingly incontrovertible point that person-level research should use person-sensitive conceptualization and measurement leads us into some thorny issues. It seems to imply that person-levelness (or person sensitivity) is an ideal that is always imperfectly realized rather than a dichotomous property. This in turn suggests that the three branches are not as discrete as they might seem. In this commentary, I elaborate on this problem and its implications.

## Person-Sensitive Conceptualization and Measurement

Person level research aspires toward “understanding and explaining psychological phenomena as experienced and enacted by individual persons”, according to Lundh (2023, p.76). It is important to note that not all methods that are employed within the person-oriented tradition are optimally conductive to this goal. Although the focus of person-oriented research is on within-person patterns that are assumed to represent the systems and developmental trajectories under scrutiny (Bergman & Trost, 2006; Magnusson,^1999^), the units of the patterns—that which they varyacross—are typically scores *on variables* that were not de-signed to capture individuality. In fact, the appropriateness of the variables is often justified in terms of evidence procured through factor analysis and other kinds of population-based statistics (e.g., Sica & Sestito, 2020; Travis & Craig, 2023), which capture systematic differences *between* individuals rather than properties *within* individuals (Lamiell,^1987^; Molenaar, 2004). Therefore, while these methods areclearly *person-oriented* in the traditional sense, they are not unequivocally at the *person level* on the broader definition that is introduced in the target article. This is *not* to say thatthese methods are illegitimate. On the contrary, I will propose that they can to some extent be useful for procuringknowledge about individuals even though they involve population-based statistics. Nevertheless, if we want a more complete understanding of psychological phenomena as they are experienced and enacted by individuals, then we need to be concerned with whether our conceptualizations and measurements are sensitive to the psychological meaning and structure of properties of individuals and their environments.

Although advances in person-oriented methodology have begun to address the shortcomings of standardized measurement (e.g., Bergman, 2017; Molenaar, 2004; Nesselroade & Molenaar, 2022), these have focused on statistical issues, including how to quantify properties of measurements (e.g., reliability or invariance) at the level of the individual. This is only half of the problem. The other half of the problem is how to determine what properties of persons and their environments are relevant for the psychological phenomena under scrutiny (as experienced and enacted by individuals) and how to measure these properties in a person-sensitive way—or, in other words, what data should be collected in the first place. This part of the problem has so far not attracted anywhere near as much attention as the statistical issues have within the person-oriented tradition.

This is without a doubt a daunting problem. Nevertheless, there are some practicable strategies for generating data that are both person-sensitive and potentially amenable to person-oriented statistical treatment that recur across other research traditions. One of these is to elicit person-sensitive constructs through open-ended questions and thereafter quantify those constructs through subject ratings or expert coding. For example, subjects may be asked to rate: elements in a topic domain in terms of personal constructs that have been elicited through open-ended questions (Kelly, 1955); personal life projects that they have listed freely on common dimensions such as importance, manageability, consistency with personal values, relations to other projects, and positive and negative affect (Little, 1983); or personal attributes that they have described in their own words in terms of relevance to different kinds of everyday situations (Cervone et al., 2001). Similarly, personal life-story narratives that are elicited through open-ended questions about past events, challenges in life, future aspirations, and other themes may be quantitatively coded by experts in terms of common affective, motivational, and structural elements (McAdams, 2008).

Another common strategy involves letting participants make comparative judgments. For instance, in the repertory grid technique, personal constructs are commonly elicited by having participants judge the similarity between different elements (e.g., words, phrases, or vignettes) in the topic domain under scrutiny and explain their similarities and dissimilarities (Kelly, 1955). Similarly, in Q-methodology, the participants sort a set of statements or other materials into ordered categories (e.g., from “Agree least” to “Agree most”), which forces them to compare these materials. The responses are subsequently subjected to a Q-factor analysis, and the extracted factors are interpreted qualitatively as unified subjective viewpoints, with particular focus on materials placed in the extreme categories, which are assumed to be the most subjectively significant (McKeown & Thomas, 1988). In other words, the elements of the system under scrutiny are at least to some extent individuated and defined by the subjective viewpoint as a whole rather than standardized measurement (Nilsson, 2015). In this sense, Q-methodology can be more unambiguously positioned at the person level compared to other person-oriented methods that represent the system components in terms of standardized variables.

While these types of methods have traditionally been used primarily to quantify subjectively relevant psychological properties within the person, similar strategies could be used to identify and quantify subjectively relevant aspects of the person’s environment and subjective experiences of it. This applies both to naturally occurring and manipulated environments. For example, it may be valuable to measure relevant aspects of the psychotherapeutic environment individual patients are exposed to and how they experience this environment, in line with Lundh’s (2023) points.

## The Lack of a Clear Line of Demarcation

Do these kinds of strategies for person-sensitive measurement go far enough? How do we know, for example, that the materials, dimensions, or themes that are used to collect subject or expert ratings are maximally subjectively relevant and exhaustive? Researchers with qualitative methodological sensibilities might want to object that we need more in-depth idiographic methods (e.g., ethnographical, biographical, or phenomenological) to truly understand psychological phenomena as they are experienced and enacted by individuals.

To understand psychological phenomena at the level of singular individuals *completely* may be an impossible task, at least insofar as we concede that psychological science should encompass a conception of persons as agents with mental states that exhibit intentionality and subjectivity. There are at least three fundamental philosophical reasons that understanding such mental states completely is difficult (for a more detailed description, see Nilsson, 2015):

1. *The externalism of the mental*. Intentional mental states (e.g., beliefs, intentions, emotions, and goals) derive part of their content from the aspects of the world they represent (Searle, 1983). Therefore, to understand such states we need to understand the subjective meanings of the aspects of the world they are directed at, which are shaped in idiosyncratic ways by the person’s history of causal interaction with the world.
2. *The holism of the mental*. Intentional mental states (e.g., beliefs, intentions, emotions, and goals) derive their content in part from their relations with each other (Davidson, 1970). Therefore, to understand even specific intentional states, we need an understanding of the broader systems of meaning (or worldviews) they are embedded within.
3. *The subjectivity of the mental*. Mental states have a subjective ontology—they are what they are by virtue of being experienced from the subject’s first-person perspective (Searle, 1992). Therefore, we need an understanding of the qualitative character of the subject’s experiences even though we cannot directly observe these experiences.

For all these reasons, we are necessarily relying on a plethora of idealizing assumptions (or theories of interpretation) about the mental states and environments of other individuals to make them intelligible as persons. These can always be made more nuanced and adjusted to the person’s idiosyncrasies. There is no clear point at which we can say that we have completely or perfectly understood the individual’s personal worldview, biography, and environment. How far we go in ensuring that our conclusions are sensitive to the experiences and enactments of phenomena by individuals is always going to be a matter of degree. Therefore, there is no singular line of demarcation between legitimate and illegitimate methods at the person level. Furthermore, the extent to which methods are at the person level can be conceived of as multidimensional, in the sense that methods may be more clearly at the person level in some respects than others (e.g., person-sensitive analysis, measurement, or conceptualization).

This is not just a matter of pragmatic considerations. There are also tradeoffs between different types of knowledge. Most important, the pursuit of a maximally rich idiographic understanding of individuals (e.g., through psychobiographical case studies) will inevitably place constraints on generalization. To be able to generalize, and therein benefit from sophisticated person-oriented statistical tools in the first place, we need to make assumptions about the homogeneity of mental contents and other properties across individuals—or in other words, strip away layers of subjective meaning (Nilsson, 2014). It follows from the argumentation in the preceding paragraphs that we can never completely test all these assumptions. Nevertheless, methods that are to a greater degree at the person level replace more of these assumptions with empirically based generalizations. For example, methods of generalization that rely on personoriented statistics are certainly more at the person level than traditional variable-oriented methods, in the sense that they do not make assumptions about the homogeneity of associations between variables across individuals, while methods that rely on both person-oriented statistics and person-sensitive measurement are even more at the person level, in that they do not assume that the system components can be represented by the same standardized variables.

Even methods that involve population-based statistics (e.g., to represent the system components) can to some extent be useful for procuring an understanding of individuals, albeit a crude, fallible, and probabilistic understanding with significant untested assumptions about homogeneity across individuals, because even an idealization can yield true inferences (Nilsson, 2014). In this sense, person-oriented methods that represent the system components in terms of individual difference variables can be legitimately applied at the person level. Furthermore, some population-based statistics may even strengthen the justification of inferences about individuals. For example, if the variables that are used in person-oriented studies would be shown to exhibit measurement invariance across many strata of the population, this would move us one small step in the person level direction, as this would make it reasonable to assume (or at least make an educated guess) that scores on variables are meaningfully comparable across a greater number of individuals than would be the case if there was plenty of measurement noninvariance. Similarly, even some statistical methods that are designed to identify average trends in a population (e.g., linear mixed effects and latent growth curve modeling) can move us one step in the person-level direction insofar as they allow population parameters (intercepts and slopes) to vary, which allows us to investigate the presence heterogeneity in the parameters across individuals and groups.

## Concluding Remarks

These lines of reasoning suggest that it is more appropriate to view person-levelness (or perhaps rather person-sensitivity) as an ideal we may aspire toward but can never perfectly realize than to conceive of the person level as a discrete branch of psychology. At the end of the day, what matters is that there is a critical awareness of limitations and untested assumptions of the methods we are using and an active pursuit of more person-focused descriptions and explanations when we are pursuing knowledge about individuals. In other words, we need to continually ask ourselves whether more can be done to increase our sensitivity to the participant’s individuality without unreasonable sacrifices in terms of generalizability or feasibility.

Although normative distinctions between scientific fields play an important role in stimulating critical thinking, methodological innovation, and greater coherence between assumptions, problems, and methods, there is a risk that they devolve into rigid dichotomies (e.g., “quantitative vs. qualitative”) that limit methodological creativity and impede progress within research communities that are hungry for simple rules and disciplinary identities. Cross-fertilization between person-, population-, and mechanism-focused research can be productive, as abundantly illustrated by the person-oriented research tradition, and both variable- and personoriented methods can (to varying extents) be made more person-sensitive.

## Action editor

Lars R. Bergman served as action editor for this article.

## Open access

This article is distributed under the terms of the Creative Commons Attribution 4.0 International License (http://creativecommons.org/licenses/by/4.0/), which permits unrestricted use, distribution, and reproduction in any medium, provided you give appropriate credit to the original author(s) and the source, provide a link to the Creative Commons license, and indicate if changes were made.
